# Into the headwinds: key emotional intelligence capacities that predict women's workplace wellbeing across job roles

**DOI:** 10.3389/fpsyg.2026.1848879

**Published:** 2026-05-29

**Authors:** Patricia E. Freedman, Ilaria Iseppato, Daniel Y. Choi, Joshua M. Freedman

**Affiliations:** 1 Six Seconds, Freedom, CA, United States; 2 Six Seconds, Bologna, Italy

**Keywords:** conservation of resources, emerging leaders, emotional intelligence, middle management, professional development, resilience, women leaders skills, workplace wellbeing

## Abstract

**Introduction:**

Despite decades of organizational investment, women's advancement and workplace wellbeing remain at risk. The emotional intelligence (EQ) capacities that support both have not been examined across job roles at scale. This study identifies which EQ capacities differentiate across women's career levels, are associated with Wellbeing, and how they combine across the career hierarchy.

**Methods:**

This study analyzed data from the Six Seconds Emotional Intelligence Assessment (SEI) for 43,080 women across 114 countries (2019–2025), balanced across three global regions (*n* = 14,360 each) and four career levels (Student, Employee, Management, Senior Executive). Analyses included tercile-based odds ratio analysis, temporal trajectory analysis, within-generation replication (Millennials, *n* = 21,283), and cross-regional replication.

**Results:**

Five Brain Talents (Commitment, Resilience, Proactivity, Imagination, and Risk Tolerance) formed a Resource Caravan, differentiating career levels (*d* = 0.56–0.84), while other Brain Talents showed near-zero differentiation. All three-talent combinations from the Caravan predicted Wellbeing (odds ratio [OR] = 5.12–8.62). A Resource Triad of Commitment, Resilience, and Proactivity produced the strongest effect: top-tercile women were 8.62 times more likely to report High Wellbeing (95% confidence interval [CI] [7.96, 9.34]). When Triad scores were high, Wellbeing converged across career levels, with career-level differences substantially narrowing. Women in management were the most vulnerable group, showing the weakest Triad effect (OR = 7.29) and the largest Wellbeing decline among workforce participants (Δ = −1.57). The pattern held across all 7 study years. Within-generation replication indicated that age was not the primary driver. Cross-regional replication confirmed the pattern across Asia, Europe, and North America (ORs = 7.13–9.52).

**Discussion:**

These findings suggest that targeted development of specific emotional intelligence resources may support Wellbeing for women leaders across career levels.

## 1 Introduction

Despite decades of organizational investment and research (Eagly and Carli, 2007; Heilman, 2012), women's advancement and their wellbeing at work remain at risk. Research has documented persistent structural barriers across women's career levels, including sponsorship gaps, flexibility penalties, disproportionate caregiving burdens, emotional labor demands, and promotion bias (Hochschild, 1983; Kossek et al., 2017; Landivar et al., 2020; Vial and Cowgill, 2022). But the commitment inside organizations to address these barriers is declining (McKinsey Company LeanIn.Org, 2025). Less examined are the internal capacities that contribute to both advancement and wellbeing. Emotional intelligence (EQ) has been linked to both leadership effectiveness and sustained wellbeing at work, yet a global decline in EQ from 2019 to 2024 has been documented in a pattern termed the “Emotional Recession” (Freedman et al., 2025). Identifying which specific EQ capacities support women's wellbeing across job roles is the focus of the present study.

A substantial body of scholarship has established that workplaces are not gender-neutral environments. Organizational structures, norms, and processes systematically shape women's career trajectories in ways that individual effort alone cannot overcome (Acker, 1990; Stamarski and Son Hing, 2015). Gendered patterns in hiring, evaluation, sponsorship, and promotion create unequal access to the conditions that support career development and wellbeing (Eagly and Carli, 2007; Son Hing et al., 2023). These barriers fall unevenly, with women of color, women with caregiving responsibilities, and women in precarious employment facing compounded disadvantage (Crenshaw, 1989; Stamarski and Son Hing, 2015). The present dataset does not permit disaggregation by these dimensions, but any interpretation of the findings reported here should be read with this context in mind.

Women's workplace wellbeing has two components: the capacities women bring and the conditions organizations provide (Ely and Meyerson, 2000). If some EQ capacities depend on those conditions to develop, then identifying which ones matter most for wellbeing tells organizations where to invest. The present study identifies which capacities to target.

EQ is a set of learnable emotional and cognitive capacities that enable people to integrate thinking and feeling to make optimal decisions (Freedman, 2010). These capacities are among the most consistent predictors of leadership effectiveness. Meta-analytic evidence has established that higher EQ predicts job performance, leadership emergence, and adaptive decision-making (O'Boyle et al., 2011; Miao et al., 2017). EQ also helps buffer occupational stress and preserve wellbeing under volatile conditions (Kotsou et al., 2019). If these capacities are unevenly distributed across women's career levels, the implications extend beyond individual wellbeing to the organizational systems that depend on a functioning leadership pipeline.

The emotional intelligence literature has debated whether EQ is best understood as a global factor or as a set of distinct, differentially important facets. Ability models emphasize a hierarchical structure in which specific branches contribute to a general EQ factor (Mayer et al., 2016). Trait models treat emotional self-perceptions as a coherent personality disposition measurable through self-report (Petrides et al., 2016). Both traditions have linked EQ to workplace outcomes, including leadership effectiveness and wellbeing (Miao et al., 2017). Yet this research has predominantly examined EQ as a single predictor, asking whether people with higher emotional intelligence fare better. Less is known about which specific EQ-related capacities matter most for women's wellbeing across organizational levels or whether these capacities operate cumulatively. The present study shifts the question from whether emotional intelligence predicts wellbeing to how specific capacities combine across career roles.

The Six Seconds Emotional Intelligence Assessment (SEI) is a validated 77-item self-report instrument used in over 22 languages across 169 countries (Dijk and Freedman, 2007; Freedman et al., 2005). The SEI yields scores on eight core EQ competencies and 18 Brain Talent capacities. Brain Talents are behavioral applications of the core competencies, capturing how individuals put EQ into action. They are distinct from the eight competencies themselves, representing an applied layer of the model (Freedman, 2010; Iannoccari et al., 2024).

Among the 18 Brain Talents, 5 correspond to constructs linked in prior research to leadership effectiveness, career advancement, and workplace wellbeing: Commitment, Resilience, Proactivity, Imagination, and Risk Tolerance (Zaccaro, 2007; Luthans and Youssef-Morgan, 2017; see Section 4.1 for detailed construct mapping). Definitions of all 18 Brain Talents are provided in Supplementary Table S1.

The SEI also yields scores on four Success Factors, self-reported life outcomes that reflect the broader impact of emotional intelligence on functioning (Freedman et al., 2005). Among these, Wellbeing, defined as the capacity to sustain optimal energy and functioning, was selected as the primary outcome variable for this study. Wellbeing was consistently the lowest-scoring Success Factor in global SEI data (Freedman et al., 2025), and the relationship between specific Brain Talent capacities and Wellbeing has not been examined across career roles for women. Throughout this paper, capitalized “Wellbeing” refers to this SEI-measured construct, while lowercase “wellbeing” refers to the broader concept in the research literature.

Conservation of Resources (COR) theory (Hobfoll, 1989; Hobfoll et al., 2018) provides the theoretical framework for this study. According to COR, individuals strive to obtain, retain, protect, and build resources, defined as conditions, personal characteristics, or energies that are valued or that serve as means to obtain valued ends. A central prediction is that resource dynamics are self-reinforcing: individuals with fewer resources are more vulnerable to further loss, while those with more resources are better positioned for further gain, producing what Hobfoll terms “loss spirals” and “gain spirals.” COR also predicts that resources do not travel alone but tend to aggregate in “caravans,” such that individuals who possess one valued resource are likely to possess others (Hobfoll, 2002, 2011). Organizational conditions serve as “resource passageways” that either facilitate or obstruct resource accumulation (Hobfoll et al., 2018).

In this framework, Brain Talents are personal resources: learnable capacities that contribute to career effectiveness and support wellbeing. Job role functions as a variable organizational condition that gives access to autonomy, sponsorship, and decision-making authority, each of which facilitates further resource development (Hackman and Oldham, 1976; McKinsey Company LeanIn.Org, 2025). COR thus connects EQ capacities to organizational conditions, framing personal resources not as individual traits but as capacities shaped by the environments in which women work.

This study addresses three research questions. First, which Brain Talent capacities are different across women's career levels, and how significant are these differences? Second, which Brain Talents predict Wellbeing, and do the capacities that differentiate job roles overlap with the capacities that predict Wellbeing? Third, do Brain Talent capacities interact cumulatively in their relationship to Wellbeing, and does this cumulative relationship vary across the career hierarchy?

## 2 Materials and methods

### 2.1 Participants

This study analyzed data from 43,080 women drawn from the larger 276,972-participant global database of the Six Seconds Emotional Intelligence Assessment (SEI). A stratified random sampling method ensured balanced representation across three global regions (Asia, Europe, and North America; *n* = 14,360 each), using continental boundaries that broadly align with the United Nations M49 statistical classification for standard regional grouping (United Nations Statistics Division, 2023). These regional groupings were used for replication testing, not as proxies for cultural equivalence; within-region variation in language, labor markets, and institutional contexts is substantial. Data were collected between 2019 and 2025.

Participants were classified into four career levels based on self-reported job role: Student (*n* = 3,783), Employee (*n* = 20,673), Management (*n* = 13,371), and Senior Executive (*n* = 5,253), yielding an analytic sample of 43,080 women. Entrepreneurs (*n* = 2,616) were excluded from the analytic sample because entrepreneurial roles do not map onto a hierarchical career-level framework (Lazear, 2005). Table 1 presents the demographic characteristics of the sample.

**Table 1:** Demographic characteristics of the sample.

Career level	*N*	% of sample (%)	Mean age	SD
Student	3,783	8.8	21.9	5.2
Employee	20,673	48.0	36.3	10.1
Management	13,371	31.0	41.5	8.8
Senior executive	5,253	12.2	44.4	9.4
Total	43,080	100	37.7	10.9

### 2.2 Procedure

Participants completed the SEI as part of applied practice settings, including training, coaching, organizational development programs, selection, onboarding, wellbeing and community initiatives, and other learning contexts such as higher education and personal development. Prior to accessing the assessment, all individuals were required to review and accept the instrument's terms of use and privacy policy, which stated that responses may be stored in anonymized form for future research. These terms align with international data protection standards, including Regulation (EU) 2016/679, the General Data Protection Regulation (GDPR; European Parliament Council, 2016).

All response data were collected through a secure digital platform. Personally identifiable information was either never collected or was removed during processing to ensure complete anonymization. For this study, de-identified records were extracted retrospectively and aggregated for analysis without any access to individual-level identifiers or direct participant contact.

Dataset preparation followed a standardized preprocessing protocol, which included excluding test entries, statistical outliers, and records missing essential demographic variables needed for stratified sampling. Stratification and demographic balancing procedures are detailed in Section 2.1 (Participants).

### 2.3 Materials

The Six Seconds Emotional Intelligence Assessment (SEI) is a 77-item self-report instrument that measures eight core EQ competencies and 18 Brain Talent capacities (Freedman, 2010). Respondents rate items using a five-point Likert scale ranging from “strongly disagree” to “strongly agree.” Items reflect emotional and behavioral patterns, such as “If I wanted to, I could tell a friend how I usually react to stress,” “I have many people that I can fully rely on,” “I am able to predict my reactions,” “There is a logic to feelings,” and “I've effectively resolved challenges.” Items listed are paraphrased examples. Validation studies confirm adequate construct validity for both competency and outcome scales (Stillman et al., 2017; Freedman et al., 2005). Internal consistency for the present dataset was satisfactory, with Cronbach's α coefficients ranging from 0.64 to 0.81 across the SEI scales, consistent with previously published validation (Dijk and Freedman, 2007; Emmerling, 2021). SEI scores are standardized with a normative mean of 100 and a standard deviation of 15 (Freedman, 2010).

The 18 Brain Talents represent fine-grained capacities derived from the eight core competencies and capture distinct patterns of emotional and social functioning relevant to workplace performance (Freedman, 2010; Iannoccari et al., 2024). Among the SEI's four outcome measures, Wellbeing was selected as the primary outcome variable because of its direct relevance to workplace sustainability and its theoretical alignment with COR theory (Hobfoll, 1989, 2001).

### 2.4 Data analysis

**Figure 1:**
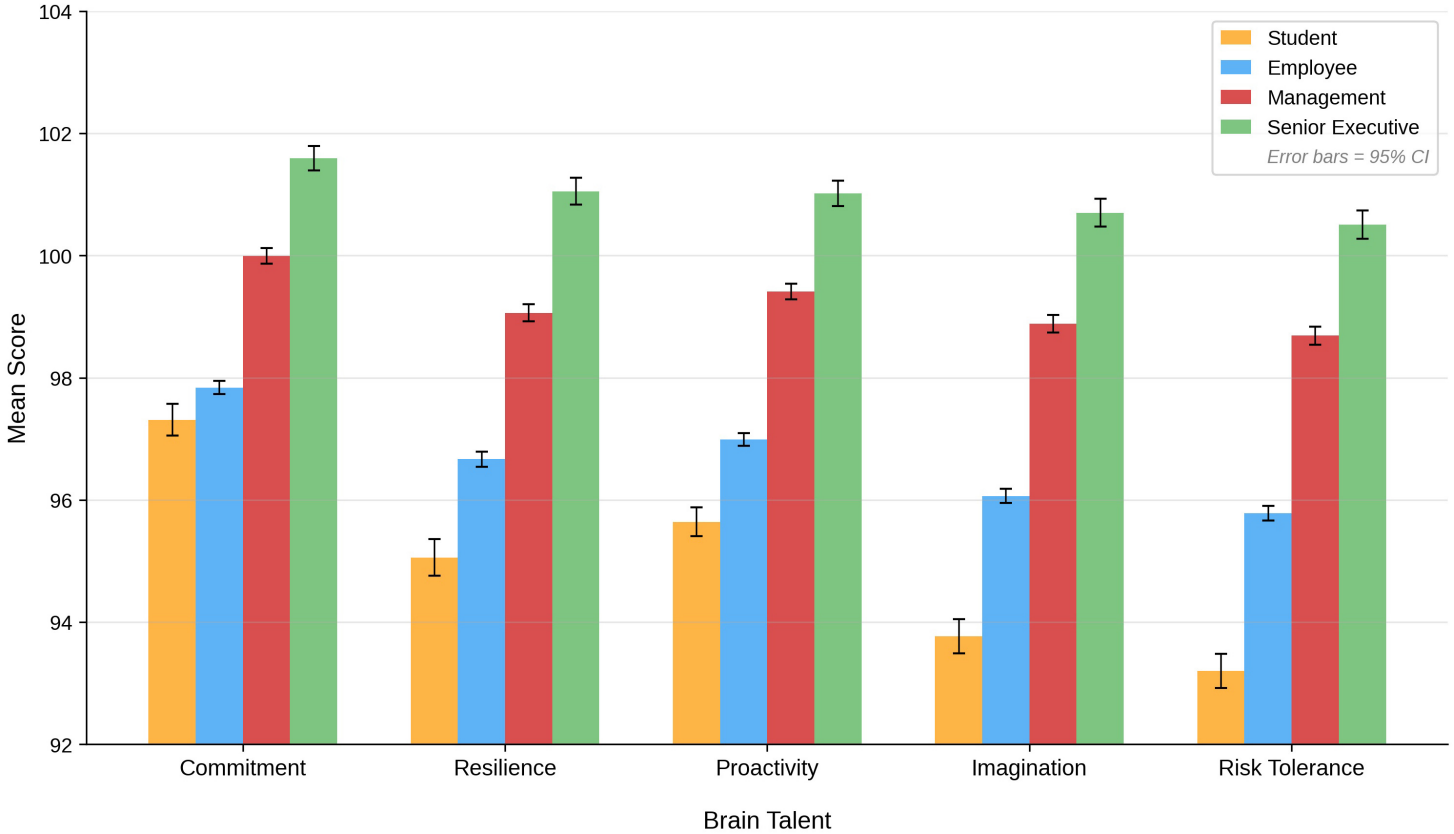
Five resource caravan brain talents by career level. All five talents show a staircase pattern with medium-to-large effect sizes (*d* = 0.56–0.84). *N* = 43,080.

**Figure 2:**
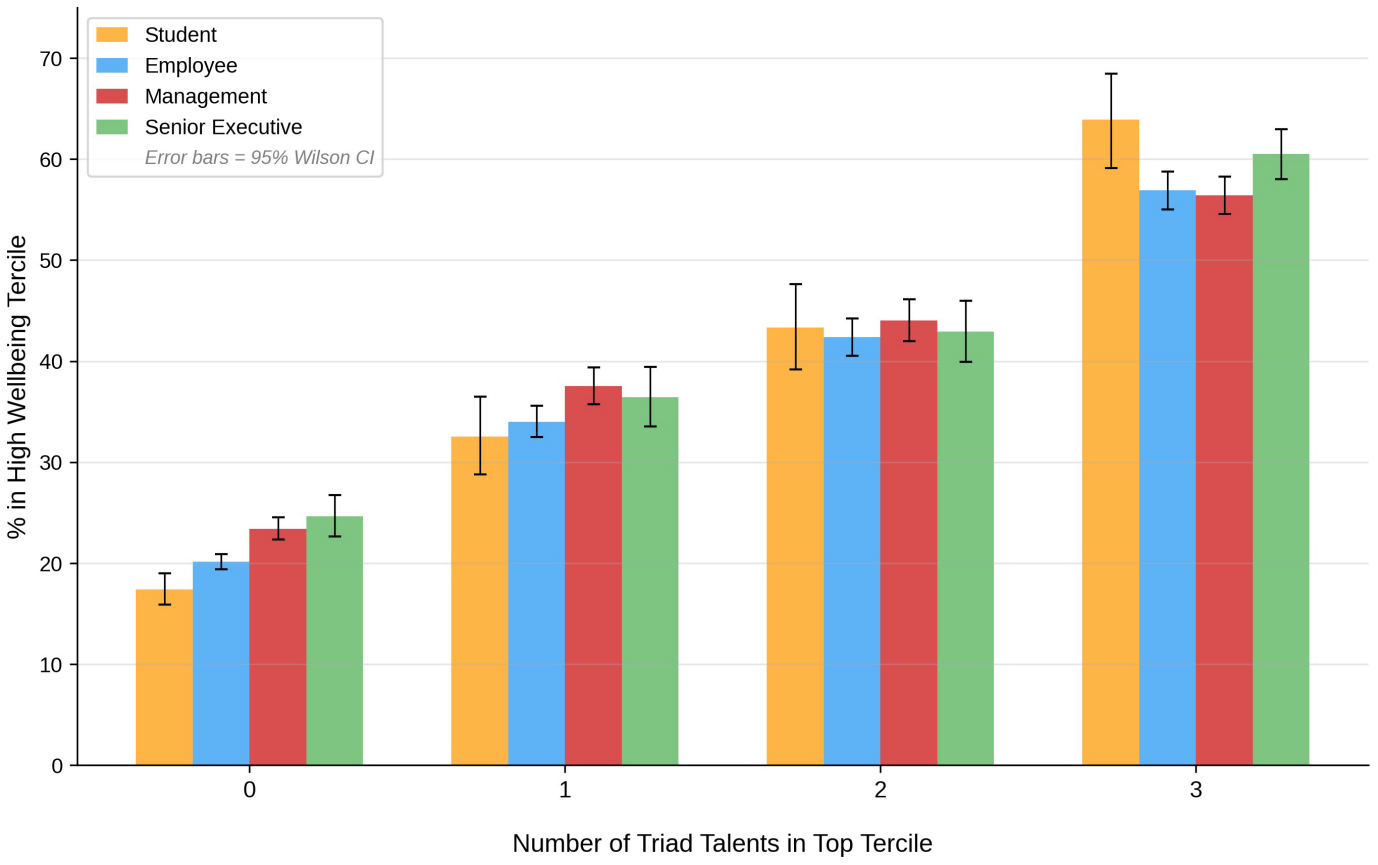
Percentage in high wellbeing tercile by career level and resource triad count. At three of three triad talents in the top tercile, career-level differences in wellbeing substantially narrow.

**Figure 3:**
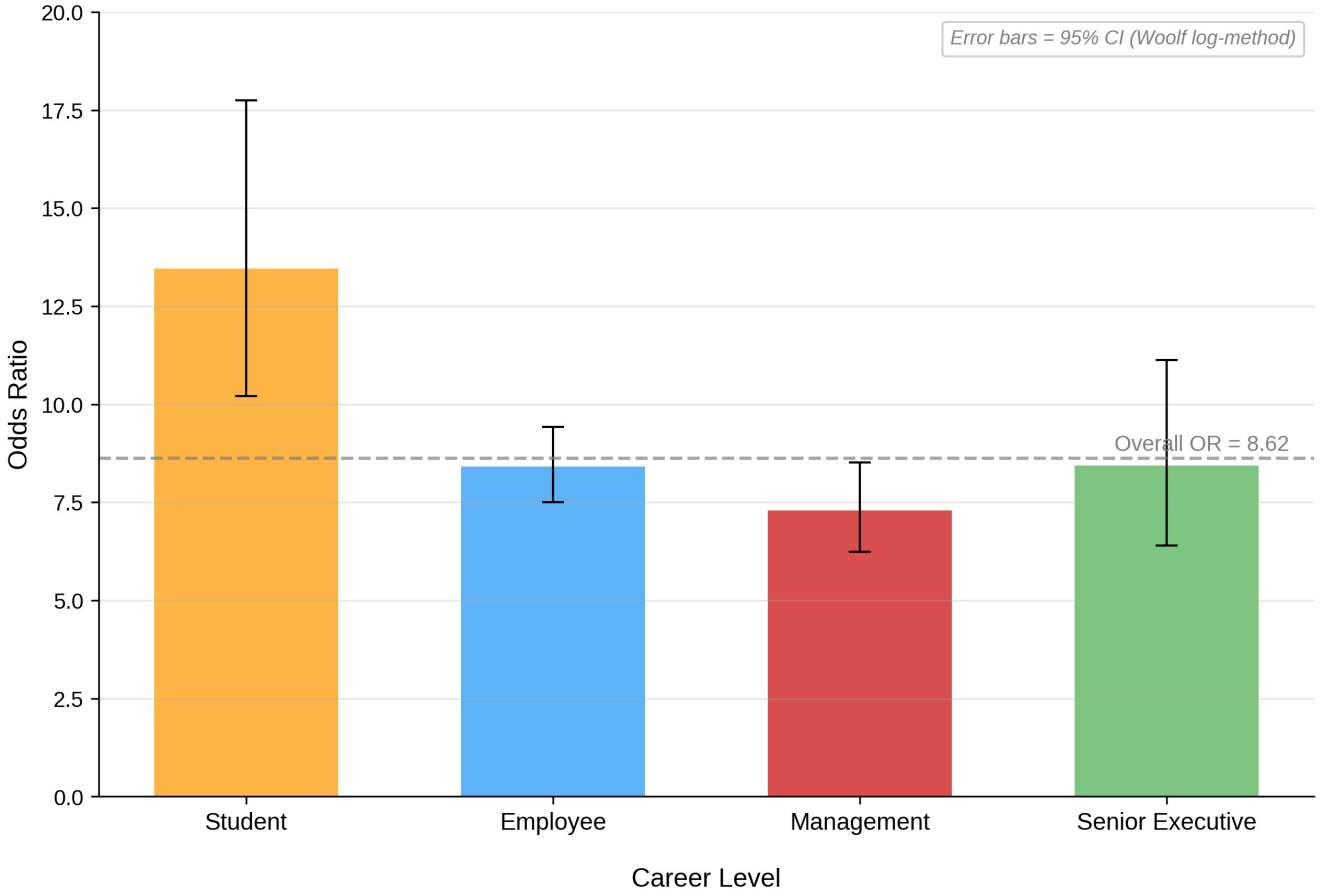
Resource triad odds ratio (OR) for high wellbeing by career level. Management women (highlighted) show the weakest triad effect (OR = 7.29). Dashed line indicates overall OR = 8.62.

**Figure 4:**
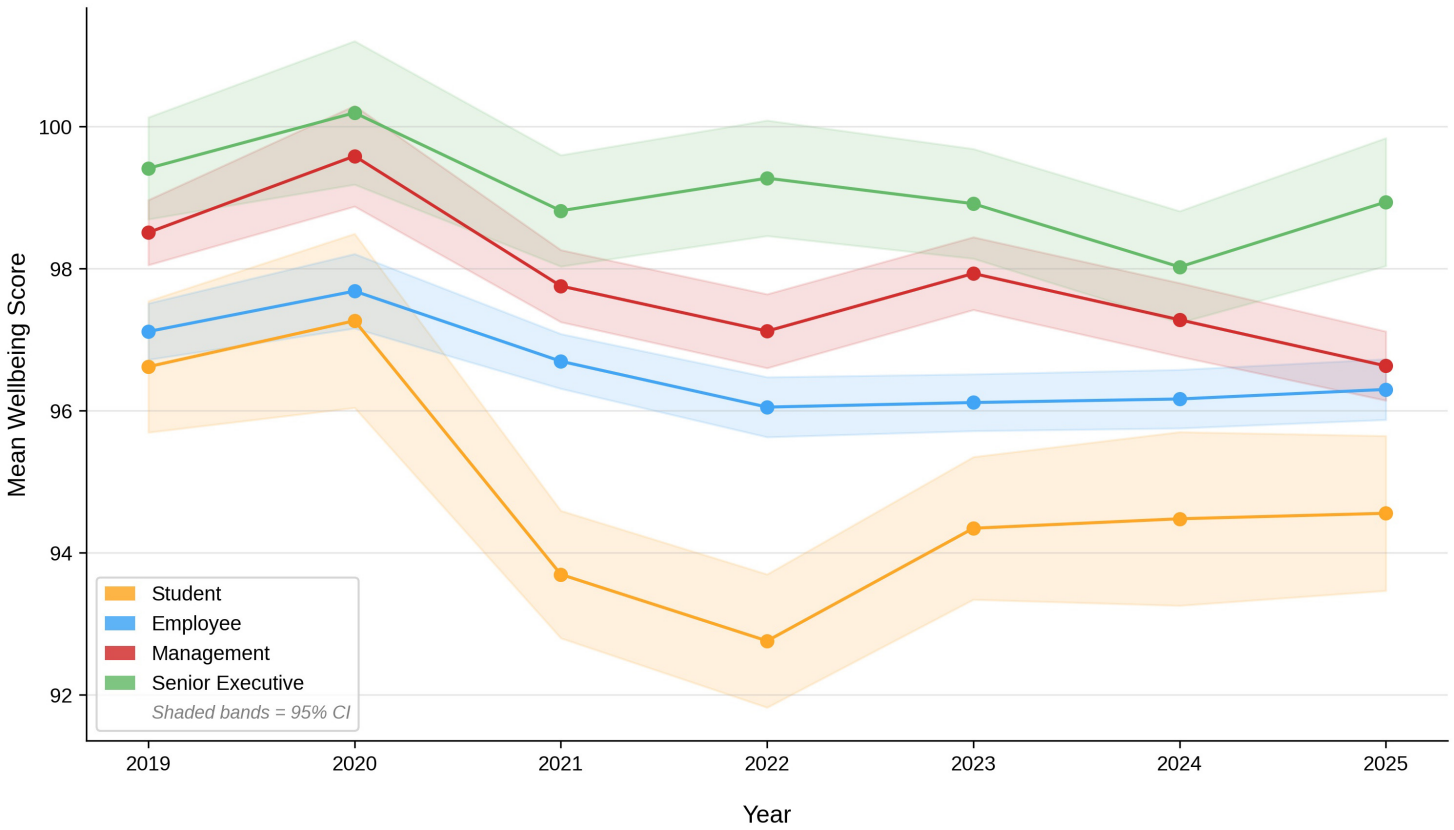
Mean wellbeing score by career level, 2019–2025. Management women show the largest decline among workforce participants (Δ = −1.57, comparing 2019 to pooled 2024–2025).

**Figure 5:**
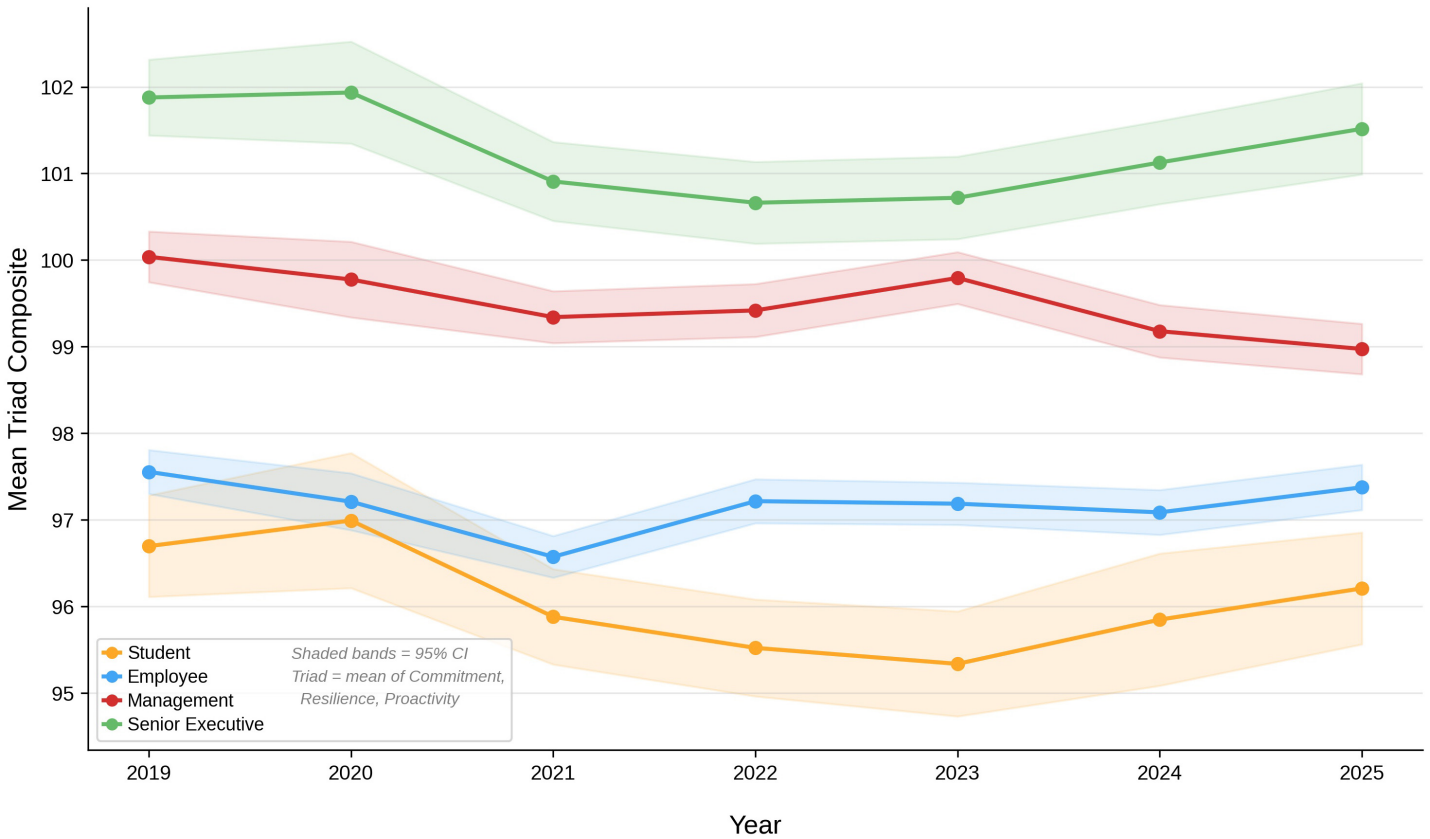
Mean resource triad composite score by career level, 2019–2025. Management women show the largest triad erosion (Δ = −1.06); none of the three individual triad talents recovered to 2019 levels.

All statistical analyses were conducted using Python 3.11 (Van Rossum and Drake, 2023) with NumPy (Harris et al., 2020) and SciPy (Virtanen et al., 2020), and independently replicated in R version 4.3 (R Core Team, 2023). Error bars and shaded ribbons in Figures 1–5 represent 95% confidence intervals; exact values are provided in Supplementary Table S3.

One-way Analysis of Variance (ANOVA) was used to examine whether Brain Talent scores differed across career levels. Effect sizes were computed using eta-squared (η^2^) for omnibus tests and Cohen's *d* for pairwise comparisons, following standard benchmarks: small = 0.20, medium = 0.50, large = 0.80 (Cohen, 1988). Bivariate Pearson correlations assessed the association between each Brain Talent and Wellbeing.

To quantify the practical magnitude of Brain Talent–Wellbeing associations, we computed tercile-based odds ratios (Norton et al., 2018). Each Brain Talent and Wellbeing was independently divided into rank-based thirds (Low, Mid, High; *n* = 14,360 per tercile), and odds ratios compared the likelihood of High Wellbeing for women in the top vs. bottom tercile. Confidence intervals were computed using the Woolf log-method (Woolf, 1955). This tercile approach was selected over a mean-split dichotomization because it (a) avoids arbitrary threshold effects, (b) provides more stable estimates with large samples, and (c) preserves information about graded associations (Norton et al., 2018).

All 10 possible three-talent combinations from the top five Brain Talents were evaluated as an exploratory robustness check. COR theory (Hobfoll, 2011) provides the interpretive framework for why personal resources should cluster and function cumulatively, but it does not prescribe which specific three-talent combination should be strongest. The combination with the highest odds ratio was labeled the Resource Triad, and the theoretical grounding from COR theory (Hobfoll, 1989; Hobfoll et al., 2018) was applied *post hoc* to interpret why those three talents cohere. Full results for all 10 combinations are reported in Section 3.5 and Supplementary Table S4 so readers can assess the full effect-size distribution rather than only the winning combination.

Robustness analyses included the following: (a) within-generation analysis restricting the sample to Millennials (born 1981–1996; *n* = 21,283) to disentangle career level from generational confounds, (b) temporal stability analysis comparing two non-overlapping time periods (2019–2021 vs. 2022–2025), and (c) cross-regional replication across Asia, Europe, and North America (*n* = 14,360 each).

To examine temporal dynamics, we computed annual mean Wellbeing scores and annual mean Resource Triad composite scores (the unweighted average of Commitment, Resilience, and Proactivity) for each career level across the 7-year study window (2019–2025). Change scores (Δ) were computed as the difference between the 2019 annual means and the pooled 2024–2025 means for each career level. The two most recent years were pooled to produce annual subsamples more comparable in size to the 2019 baseline, following the comparison window used for the temporal stability analysis.

To assess multicollinearity among the 18 Brain Talents, we computed variance inflation factors (VIF) from a multiple regression predicting Wellbeing. The conventional threshold for VIFs is 5–10 (O'Brien, 2007; Hair et al., 2010), and VIFs in the full 18-predictor model exceeded 100 for all predictors, justifying a bivariate rather than multivariate analytic strategy. For the five-predictor and Resource Triad models, VIF diagnostics are reported in Section 3.2 and Supplementary Table S5.

### 2.5 Common-method variance and multicollinearity diagnostics

We evaluated common-method variance (Podsakoff et al., 2003) by conducting a Harman's single-factor test at the item level on the 62 raw SEI items comprising the five Brain Talent predictors and the four Wellbeing outcome items. Item-level responses were retrieved from the Six Seconds SEI norm dataset for 41,926 of the 43,080 analytic-sample respondents (97.3%); item-level responses for the remaining 1,154 respondents were not retrievable from the current research export. The 1,154 unretrievable respondents did not differ meaningfully from the 41,926 retrievable respondents on any measured Brain Talent or Wellbeing variable (all |Cohen's *d*| ≤ 0.09), nor on Job Role or Region (Cramér's *V* < 0.02); 95.3% of the unretrievable respondents were from 2025, reflecting the SEI norm dataset's export window rather than a selection mechanism. Items were reverse-coded per the SEI scoring specification before extraction. Multicollinearity was assessed via variance inflation factors (VIF) for the full five-predictor model and for supplementary models dropping either Imagination or Risk Tolerance. The Resource Triad model (Commitment, Resilience, and Proactivity), which carries the paper's central analyses, was tested separately. All computations were performed in R (unrotated principal components analysis via prcomp; VIF via car::vif; Fox and Weisberg, 2019) and independently reproduced in Python.

### 2.6 Ethics approval

This study was approved by the Heartland Institutional Review Board (HIRB project No. 616-102224). Informed consent was obtained from all participants in accordance with institutional ethical standards. The data were originally collected through the SEI questionnaire for non-research purposes such as coaching, training, team selection, and personal development. Under U.S. federal regulations, the use of these de-identified educational assessment results qualifies as exempt research per HHS §46.104(d)(2)(i).

## 3 Results

### 3.1 Descriptive statistics

Brain Talent mean scores ranged from 97.03 (Risk Tolerance, *SD* = 8.90) to 100.49 (Prioritizing, *SD* = 8.06). All 18 Brain Talents showed approximately normal distributions with skewness values between −0.51 and 0.08 and kurtosis values between −1.04 and −0.60, well within conventional thresholds for normality (George and Mallery, 2010). Wellbeing (*M* = 97.03, *SD* = 11.68) also showed normal distribution.

The Wellbeing tercile cutpoints, following the rank-based thirds approach (Norton et al., 2018), were: Low [75.00, 91.61], Mid [91.61, 103.66], and High [103.66, 118.91] (*n* = 14,360 per tercile). Table 2 presents Wellbeing descriptive statistics by career level.

**Table 2:** Wellbeing by career level.

Career level	*N*	M	SD	% High WB (%)
Student	3,783	94.59	12.21	25.5
Employee	20,673	96.53	11.61	30.5
Management	13,371	97.70	11.57	34.2
Senior executive	5,253	99.03	11.37	38.1
Overall	43,080	97.03	11.68	33.3

### 3.2 Common-method variance and multicollinearity

The Harman's single-factor test yielded a first unrotated factor accounting for 19.86% of the total item-level variance (12 factors with eigenvalue ≥ 1), well below the 50% threshold that would indicate a single common factor accounts for the observed associations. Predictors and outcome are measured by distinct item sets within the SEI (58 competency items for Brain Talent predictors; 4 non-overlapping items for Wellbeing), the predictor–outcome bivariate correlations are moderate (*r* = 0.306–0.345), and the Resource Triad findings replicate across three independent regional subsamples and two non-overlapping time periods. Two supplementary variants (52 non–Positive Impression EQ items + 4 Wellbeing items = 21.42%; 58 EQ + 19 all-outcome items = 21.04%) produced comparable results. Variance inflation factors in the full five-predictor model were severe for Imagination (VIF = 169.18) and Risk Tolerance (VIF = 169.77), reflecting the *r* = 0.982 correlation between these two Brain Talents (see Supplementary Table S5). Supplementary models that drop either talent reduced all VIFs to below 10 (maximum 9.22) with negligible change in predictive performance (*R*^2^ = 0.1571 and 0.1573 vs. 0.1573 for the full model), indicating the two talents are statistically interchangeable. For the Resource Triad model (Commitment + Resilience + Proactivity) that anchors the analyses in Table 3 and Figures 2, 3, and 5, all VIFs were below 5 (maximum 4.62), within conventionally acceptable limits (Hair et al., 2010).

**Table 3:** All 10 three-talent combinations: tercile-based odds ratios for wellbeing.

Three-talent combination	OR	CI lower	CI upper
Commitment + resilience + proactivity	8.62	7.96	9.34
Resilience + proactivity + risk tolerance	8.01	7.40	8.66
Resilience + proactivity + imagination	7.93	7.33	8.56
Commitment + resilience + risk tolerance	7.60	7.04	8.21
Commitment + resilience + imagination	7.08	6.58	7.62
Commitment + proactivity + imagination	6.35	5.92	6.82
Commitment + proactivity + risk tolerance	6.31	5.88	6.78
Commitment + imagination + risk tolerance	5.91	5.52	6.32
Resilience + imagination + risk tolerance	5.80	5.42	6.20
Proactivity + imagination + risk tolerance	5.12	4.80	5.46

### 3.3 Brain talent differentiation across career levels

One-way ANOVA revealed significant career-level differences for all 18 Brain Talents (all *p* < 0.001). However, effect sizes varied substantially, identifying a clear separation between talents sensitive to career level and those that are not.

Five Brain Talents showed the steepest career-level gradients, all representing medium-to-large effects (Cohen, 1988): Risk Tolerance (η^2^ = 0.055, *d* = 0.84), Imagination (η^2^ = 0.053, *d* = 0.82), Proactivity (η^2^ = 0.044, *d* = 0.71), Resilience (η^2^ = 0.039, *d* = 0.69), and Commitment (η^2^ = 0.033, *d* = 0.56). All pairwise comparisons between adjacent career levels were statistically significant for the top five talents (all *p* < 0.001). The remaining 13 Brain Talents showed substantially smaller career-level differentiation (η^2^ = 0.002–0.028). Table 4 presents the full results.

**Table 4:** Career-level differentiation for all 18 brain talents.

Brain talent	Student	Employee	Management	Senior exec.	η^2^	*d*
Risk Tolerance^*^	93.72	96.45	98.85	101.61	0.055	0.84
Imagination^*^	93.78	96.50	98.90	101.17	0.053	0.82
Proactivity^*^	94.36	96.62	98.93	101.18	0.044	0.71
Resilience^*^	95.02	97.34	99.30	101.57	0.039	0.69
Commitment^*^	95.88	97.78	99.17	100.90	0.033	0.56
Vision	96.47	98.10	99.50	101.40	0.028	0.50
Entrepreneurship	96.19	97.85	99.67	101.58	0.027	0.49
Designing	97.11	98.41	99.56	101.05	0.018	0.39
Problem solving	97.53	98.79	99.81	101.14	0.015	0.35
Critical thinking	97.89	99.24	100.07	100.85	0.011	0.29
Adaptability	98.11	99.15	99.89	100.92	0.009	0.27
Data mining	99.01	99.43	100.10	100.87	0.005	0.18
Prioritizing	99.52	100.21	100.60	101.05	0.004	0.15
Collaboration	99.55	99.82	100.22	100.59	0.003	0.13
Modeling	99.81	100.05	100.25	100.41	0.002	0.06
Emotional insight	99.62	99.78	100.15	100.92	0.002	0.13
Reflecting	100.08	99.98	100.01	100.09	0.001	0.01
Connection	99.71	99.85	100.08	100.31	0.001	0.06

Values are means. ^*^ = top five Resource Caravan talents. d = Cohen's d for Student vs. Senior Executive comparison. All F-tests p <0.001.

### 3.4 Brain talents as predictors of wellbeing

The same five talents showing the steepest career-level gradients were also the strongest Wellbeing predictors. Commitment showed the highest bivariate correlation with Wellbeing (*r* = 0.345, 95% CI [0.337, 0.354]), followed by Resilience (*r* = 0.338), Proactivity (*r* = 0.330), Imagination (*r* = 0.319), and Risk Tolerance (*r* = 0.306). This overlap across two independent analyses is the central pattern of the study.

Women in the top tercile of each Brain Talent were substantially more likely to be in the top Wellbeing tercile than women in the bottom tercile. Tercile-based odds ratios (Norton et al., 2018; Woolf log-method CIs; Woolf, 1955) for the top five were as follows: Commitment (OR = 4.31, 95% CI [4.08, 4.54]), Resilience (OR = 4.21, 95% CI [3.99, 4.44]), Proactivity (OR = 3.95, 95% CI [3.75, 4.16]), Imagination (OR = 3.62, 95% CI [3.43, 3.81]), and Risk Tolerance (OR = 3.40, 95% CI [3.23, 3.58]). Table 5 presents the full results.

**Table 5:** 18 Brain talent–wellbeing associations: correlations and tercile-based odds ratios.

Brain talent	*r*	OR	95% CI lower	95% CI upper
Commitment^*^	0.345	4.31	4.08	4.54
Resilience^*^	0.338	4.21	3.99	4.44
Proactivity^*^	0.330	3.95	3.75	4.16
Imagination^*^	0.319	3.62	3.43	3.81
Risk tolerance^*^	0.306	3.40	3.23	3.58
Vision	0.289	3.12	2.96	3.29
Entrepreneurship	0.274	2.89	2.74	3.04
Designing	0.255	2.67	2.53	2.81
Problem solving	0.243	2.52	2.39	2.66
Critical thinking	0.221	2.29	2.17	2.41
Adaptability	0.209	2.15	2.04	2.27
Data mining	0.182	1.92	1.82	2.02
Prioritizing	0.165	1.78	1.69	1.88
Collaboration	0.148	1.65	1.57	1.74
Modeling	0.131	1.53	1.45	1.61
Emotional insight	0.119	1.44	1.37	1.52
Reflecting	0.098	1.32	1.25	1.39
Connection	0.087	1.25	1.19	1.32

*Top five Resource Caravan talents. OR = tercile-based odds ratio (top vs. bottom third). CI = Woolf log-method 95% confidence interval.

### 3.5 Cumulative effects: the resource triad

All 10 possible three-talent combinations from the top five Brain Talents produced significant Wellbeing effects, with odds ratios ranging from 5.12 to 8.62 (Table 3). The strongest combination, Commitment, Resilience, and Proactivity, was designated the Resource Triad (OR = 8.62, 95% CI [7.96, 9.34]). Women in the top tercile of all three Triad talents were 8.62 times more likely to report High Wellbeing than women in the bottom tercile. This odds ratio reflects a two-tercile shift on each talent simultaneously, comparing the upper and lower tails of the joint distribution rather than a one-unit shift on a continuous scale; the magnitude should be interpreted in this context.

Combinations containing both Commitment and Resilience averaged an OR of 7.77, compared to 6.49 for combinations missing at least one (bootstrap-verified mean difference = 1.28, 95% CI [0.99, 1.43], *p* < 0.05), identifying Commitment and Resilience as anchor resources within the caravan.

The Resource Triad effect held across all four career levels: Student (OR = 13.46), Employee (OR = 8.41), Management (OR = 7.29), and Senior Executive (OR = 8.44). Table 6 presents the career-level breakdown.

**Table 6:** Resource triad odds ratios by career level.

Career level	*N*	OR	CI lower	CI upper
Student	3,783	13.46	10.21	17.74
Employee	20,673	8.41	7.56	9.36
Management	13,371	7.29	6.41	8.28
Senior executive	5,253	8.44	6.80	10.47
Overall	43,080	8.62	7.96	9.34

The Resource Triad effect was graded rather than threshold-based. Among women in the bottom tercile on all three Triad talents, 21.1% were in the High Wellbeing tercile. Each additional top-tercile talent increased the proportion by approximately 12 percentage points: one talent (35.4%), two talents (43.1%), three talents (57.8%). This graded pattern held across all four career levels (Table 7).

**Table 7:** Percentage of women in high wellbeing tercile by triad count and career level.

Triad count	Student (%)	Employee (%)	Mgmt (%)	Senior exec. (%)	Overall (%)
0 of 3	17.4	20.1	23.4	24.6	21.1
1 of 3	32.5	34.0	37.5	36.4	35.4
2 of 3	43.3	42.4	44.0	42.9	43.1
3 of 3	63.9	56.9	56.4	60.5	57.8

At zero Triad talents, career level produced the expected spread in Wellbeing outcomes (Student: 17.4%, Employee: 20.1%, Management: 23.4%, Senior Executive: 24.6%; χ^2^_(3)_ = 56.46, *p* < 0.001, Cramér's *V* = 0.052). At three Triad talents, the staircase narrowed substantially (56.4% to 63.9%, spread 7.5 pp; χ^2^_(3)_ = 13.73, *p* = 0.003, Cramér's *V* = 0.043) and the rank order shifted—Students and Senior Executives scoring high on all three Triad talents showed the highest High Wellbeing rates (63.9% and 60.5%), with Employees and Managers slightly below (56.9% and 56.4%). A pairwise comparison of the lowest and highest career levels at zero Triad talents (Student vs. Senior Executive) was highly significant (*z* = −5.61, *p* < 0.001), whereas the same pairwise comparison at three Triad talents did not reach significance (*z* = 1.24, *p* = 0.21). Career level's predictive power weakened as personal resources accumulated, though it did not fully disappear.

### 3.6 Disentangling age and career-level effects

*Within-generation analysis*. Because career level is partially confounded with age, we restricted the sample to Millennials (born 1981–1996; *n* = 21,283), the only generation spanning all four career levels in sufficient numbers. The Resource Triad OR within this generational cohort was 8.94 (95% CI [8.01, 9.99]), closely approximating the full-sample estimate of 8.62. This within-generation replication suggests that the observed career-level stratification reflects role-related processes rather than generational differences.

*Temporal stability*. To assess the stability of the Resource Triad effect, we divided the dataset into two non-overlapping periods: 2019–2021 (*n* = 19,847) and 2022–2025 (*n* = 23,233). The Resource Triad OR remained stable across both periods (Period 1 OR = 8.48; Period 2 OR = 8.72).

*Temporal trajectories*. Mean Wellbeing scores declined across the study period for all career levels, but the magnitude and pattern of decline varied (Figure 4). Management women showed the largest Wellbeing decline among workforce participants (Δ = −1.57, *d* = −0.14), falling from *M* = 98.51 in 2019 to *M* = 96.94 in 2024–2025. Unlike other career levels, Management Wellbeing declined steadily and did not recover. Students showed a larger absolute decline (Δ = −2.10, *d* = −0.18) but from a lower baseline. Senior Executives showed the smallest decline (Δ = −0.97). The Resource Triad composite (mean of Commitment, Resilience, and Proactivity) showed a parallel pattern (Figure 5; Supplementary Table S3): Management women experienced the largest Triad decline of any career level (Δ = −1.06), and none of the three individual Triad talents (Commitment: −0.98, Resilience: −1.02, Proactivity: −1.19) recovered to 2019 levels in any subsequent year. By contrast, Employee and Senior Executive Triad scores remained relatively stable (Δ = −0.18 and −0.36, respectively).

*Cross-regional replication*. The Resource Triad association replicated across all three global regions: Asia (OR = 9.52), Europe (OR = 7.13), and North America (OR = 9.43). While absolute magnitudes varied, likely reflecting regional differences in assessment contexts and emotional expression norms (Matsumoto et al., 2008), the pattern of Commitment, Resilience, and Proactivity as the strongest three-talent combination was consistent across regions. Table 8 presents the full cross-regional results.

**Table 8:** Cross-regional replication of the resource triad.

Region	*N*	OR	CI lower	CI upper
Asia	14,360	9.52	8.34	10.87
Europe	14,360	7.13	6.26	8.12
North America	14,360	9.43	8.23	10.81
Overall	43,080	8.62	7.96	9.34

Table 9 summarizes the Resource Triad odds ratios across all robustness analyses.

**Table 9:** Summary of robustness analyses.

Analysis	*N*	OR	CI lower	CI upper
Full sample	43,080	8.62	7.96	9.34
Millennials only	21,283	8.94	8.01	9.99
Period 1 (2019–2021)	19,847	8.48	—^a^	—^a^
Period 2 (2022–2025)	23,233	8.72	—^a^	—^a^
Asia	14,360	9.52	8.34	10.87
Europe	14,360	7.13	6.26	8.12
North America	14,360	9.43	8.23	10.81

^a^Period-specific confidence intervals (CIs) are not reported because tercile boundaries were computed on the full sample; within-period CIs would reflect boundary misalignment rather than true sampling variability. Stability was assessed via bootstrap (OR difference = 0.24, 95% CI spanning zero).

## 4 Discussion

Among 18 EQ capacities measured, this study identifies a small cluster linked to women's Wellbeing across job roles. Only five increased with career level and were associated with higher Wellbeing. The pattern was consistent across all 7 years of data and across three global regions, linking specific EQ capacities, career level, and Wellbeing outcomes. These data align with growing evidence that emotional intelligence and structural conditions shape women's workplace outcomes (Pirsoul et al., 2023; Heilman and Caleo, 2018), and they extend that work by showing that a specific combination of EQ capacities corresponds to substantially narrower Wellbeing differences across the career hierarchy.

Women's Wellbeing at work may be more closely associated with whether women have developed specific EQ capacities than with the resource dynamics typically associated with their career level. Conservation of Resources (COR) theory (Hobfoll, 1989; Hobfoll et al., 2018) predicts that organizational position shapes access to gain spirals and loss spirals, and the career-level patterns in this study are broadly consistent with that prediction. The exception is Managers, who occupy a position that should access gain spirals for resource advantages but who show the lowest Wellbeing levels, suggesting that position alone is not sufficient without the EQ capacities to convert that position into resources.

The five Brain Talents may also function as a resource passageway. Among women who score high on these capacities, Wellbeing outcomes are similar across career levels, regardless of the structural advantages or disadvantages their position typically confers. To interpret these patterns, the following sections draw on COR theory and triangulate the findings with large-scale industry research on women's workplace conditions.

### 4.1 The resource caravan: brain talent differentiation across job roles

In data collected from 2019 to 2025, five Brain Talents measured by the SEI (Commitment, Resilience, Proactivity, Imagination, and Risk Tolerance) increased with each successive job role, with medium-to-large effect sizes (*d* = 0.56–0.84). COR theory, applied here as an interpretive framework, suggests that these five talents may function as a “Resource Caravan”: personal resources that travel together and develop through the gain spirals that a woman's organizational position provides. Duchek et al. (2022) identified a similar developmental progression in which women build contextual resilience resources during early career stages and deploy those resources as active resilience behaviors in senior leadership positions. This pattern is reflected in the staircase observed in the data.

Each of the five Brain Talents in the Resource Caravan corresponds to a well-established construct in organizational psychology. Table 10 presents the SEI definitions and their academic parallels.

**Table 10:** Resource caravan brain talent definitions and construct mapping.

Brain talent	SEI definition (Freedman, 2014)	Academic construct parallel
Commitment	*Maintaining attention on what is important*. To maintain clarity about what matters, you take your internal drive and link it to your long-term vision.	Goal-setting theory: directing effort toward valued outcomes (Locke and Latham, 2002, 2019)
Resilience	*Bouncing ahead*. To overcome obstacles, you identify opportunities and take ownership of solutions.	PsyCap: sustaining functioning and moving beyond adversity toward renewed success (Luthans, 2002; Luthans and Youssef-Morgan, 2017)
Proactivity	*Acting based on internal drive*. To address challenges before they arise, you don't wait for others, you harness your own internal drive.	Proactive motivation: self-initiated action linked to career advancement and job performance (Fuller and Marler, 2009; Zhang et al., 2022)
Imagination	*Seeing the unknown*. To envision the unknown, you blend emotional openness with cognitive clarity.	Creative problem-solving: generating novel solutions that predict workplace innovation (Mumford et al., 2000; Mumford and England, 2022)
Risk tolerance	*Handling stress and complexity*. To handle complexity, you focus on the future potential and take charge of your emotional energy.	Tolerance for ambiguity: a core predictor of leader effectiveness (Zaccaro, 2007)

SEI, Six Seconds Emotional Intelligence Assessment.

Each of these five capacities is recognized as a requirement for leadership effectiveness (Zaccaro, 2007; Dogru, 2022). In this study, they are both the most sensitive to career level and the most predictive of Wellbeing.

Why these 5 and not others? Of the remaining 13 Brain Talents, most showed small career-level differences, and 3 showed virtually none: Connection (*d* = 0.06), Emotional Insight (*d* = 0.13), and Reflecting (*d* = 0.01) were essentially equivalent across all job roles, meaning that the majority of Brain Talents develop and persist independently of organizational position (see Table 4 for full career-level data across all 18 Brain Talents).

The five Caravan Brain Talents are the capacities most likely to require organizational conditions to exercise: sponsorship to maintain commitment, supportive environments to build resilience, psychological safety to act proactively, visibility to apply creative solutions, and autonomy to take risks. In COR terms, they are the personal resources most dependent on condition-resource access, which explains why they are the ones that increase with career level. Large-scale industry surveys confirm that these conditions are unequally distributed across career levels. Women in entry-level positions are roughly half as likely as men at their level to have senior sponsors, receive fewer stretch assignments, and are less likely to be put forward for promotion (McKinsey Company LeanIn.Org, 2025), patterns that reflect the blocked resource passageways that COR predicts.

This career-level pattern was stable across the 7-year study window and replicated across all three regions (see Tables 4, 8).

The five Caravan Brain Talents appear as a staircase seen in Figure 1, but could this be a product of maturation rather than organizational position? To test this, we examined Millennials (*n* = 21,283), a generation with substantial representation at every career level and a narrow age range across those levels (mean age 32.8 for Employee, 35.4 for Managers, 35.7 for Senior Executives). Within this cohort, the staircase persisted for all five Caravan Brain Talents (all pairwise comparisons *p* < 0.001), with effect sizes roughly half those of the full sample (*d* = 0.38–0.43). Approximately half the career-level variation in the full sample may be associated with age; the remaining half reflects genuine role-based differentiation. Two forces appear to coexist: part of the career-level variation tracks age (Roberts et al., 2006), and part persists within a narrow age band, consistent with either selection or development, which the present design cannot distinguish.

If the five Caravan Brain Talents are the personal resources most dependent on organizational conditions, then those conditions become a point of leverage. Organizations that expand these supports to women at earlier career levels may accelerate the development of the capacities that matter most for both advancement and Wellbeing. Förster et al. (2025) demonstrated that organizational-level supports are critical for women leaders to translate individual resilience into ongoing performance. The staircase suggests that these capacities can be developed; the question is whether organizations reserve that development for women who have already reached senior roles or invest in it earlier. Industry evidence suggests the window may be narrowing: only half of companies now prioritize women's career advancement, down from 90% in 2021, while companies that maintained investment saw accelerated gains in women's representation across all levels (McKinsey Company LeanIn.Org, 2025).

### 4.2 Anchor resources: commitment and resilience

The five Brain Talents that differentiate career levels are also the strongest predictors of Wellbeing, with bivariate correlations ranging from r = 0.306 (Risk Tolerance) to r = 0.345 (Commitment) and individual odds ratios from 3.40 to 4.31. The capacities most shaped by condition resources are the same capacities that are most consequential for how women are doing. COR theory's resource caravan concept predicts exactly this overlap. Resources that develop together also protect together (Hobfoll, 2002, 2011).

Research on psychological capital supports this clustering. Luthans et al. (2015) demonstrated that hope, efficacy, resilience, and optimism operate as a higher-order construct, jointly predicting work outcomes more powerfully than any single component.

What drives workplace performance also safeguards women's Wellbeing, making the Resource Caravan simultaneously a personal asset and an organizational one.

Among the five Caravan Brain Talents, Commitment and Resilience stand out. Combinations containing both averaged an OR of 7.77 for High Wellbeing, compared to an OR of 6.49 for combinations missing at least one of the two (bootstrap-verified mean difference = 1.28, 95% CI [0.99, 1.43], *p* < 0.05). Commitment and Resilience anchor the caravan, amplifying whichever third talent accompanies them. The broader pattern confirms their centrality: all 10 possible three-talent combinations drawn from the five Caravan Brain Talents produced significant Wellbeing advantages (OR range = 5.12 to 8.62), but the combinations anchored by both Commitment and Resilience consistently occupied the top of the range. Resource caravans predict that once a critical mass of resources is present, the specific composition matters less than the overall supply (Hobfoll et al., 2018). The data partially support this: any three of the five confer substantial benefit. But composition also matters, and Commitment and Resilience are the hub around which the caravan organizes.

COR predicts that certain key resources occupy a central position within caravans, enabling the acquisition and deployment of others (Hobfoll, 2011; Halbesleben et al., 2014). A woman who sustains drive toward what matters (Commitment) and overcomes obstacles by finding opportunities in adversity (Resilience) creates the conditions in which Proactivity, Imagination, or Risk Tolerance can operate effectively. Goal-setting theory positions commitment as the mechanism that directs effort toward valued outcomes and enables the pursuit of subordinate goals (Locke and Latham, 2002, 2019). Integrative reviews of resilience confirm its forward-moving quality: resilience does not simply restore baseline functioning but generates adaptive growth that supports exploration and risk-taking (Raetze et al., 2022). Together, these two capacities appear to create a foundation from which other resources can be built.

McKinsey Company LeanIn.Org (2025) provide a striking parallel: when women receive strong career support, including sponsorship, manager advocacy, and senior colleague investment, their enthusiasm for promotion rises sharply. The conditions that fuel Commitment are not dispositional; they are environmental, a point that self-determination theory reinforces through its emphasis on contexts that support or undermine intrinsic motivation (Deci and Ryan, 2000; Gagné et al., 2022). When those conditions are absent, even women with high initial drive face erosion (Hobfoll et al., 2018).

### 4.3 The resource triad: a caravan for wellbeing

The strongest combination among all 10 possible three-talent groupings was designated the “Resource Triad”: Commitment, Resilience, and Proactivity (OR = 8.62, 95% CI [7.96, 9.34]), drawing on COR theory's prediction that resources cluster and function cumulatively (Hobfoll, 2011). The effect was graded rather than all-or-nothing. Among women in the bottom tercile on all three Triad talents, only 21.1% were in the High Wellbeing tercile. Each additional talent in the top tercile added approximately 12 percentage points of Wellbeing protection: one talent raised the figure to 35.4%, two to 43.1%, and all three to 57.8% (see Figure 2). The pattern is linear and cumulative instead of threshold-based, meaning there is no magic level of Triad talents where benefits to Wellbeing suddenly engage. A woman with two of the three is substantially better off than a woman with zero, even if she lacks the full Triad. This is relevant for organizations because skill development efforts that strengthen any of the three Triad talents can improve Wellbeing outcomes (Powell et al., 2024). Women do not need to develop all three Triad talents to benefit.

The addition of Proactivity to the anchor resources of Commitment and Resilience forms the Triad. Parker et al. (2010) identified proactive motivation as the mechanism through which individuals translate internal states into self-initiated action, moving beyond what is required to what is possible. For women navigating workplaces where advancement requires self-advocacy and visible contribution (Fuller and Marler, 2009; Zhang et al., 2022), this capacity carries particular weight. Yet proactive behavior is interpersonal risk-taking, and deploying it requires psychological safety, the shared belief that risk-taking will not be punished (Edmondson, 1999; Frazier et al., 2017). McKinsey Company LeanIn.Org (2025) report that entry- and mid-level women feel less safe taking risks (71% and 73%) than senior-level women (83%), and less comfortable disagreeing with others (62% and 65%) than senior-level women (88%). This pattern parallels the Proactivity staircase in our data, implying that opportunities to act proactively may be more available at senior career levels. The Resource Triad captures the full sequence: the drive to persist (Commitment), the capacity to recover and grow (Resilience), and the willingness to act (Proactivity). Together, these three capacities account for nearly all of the Wellbeing prediction in the five-talent caravan.

The Resource Triad may also help explain how women sustain Wellbeing in the face of a demand that cuts across all career levels. Women carry a disproportionate share of emotional labor in workplace settings, the ongoing work of managing the gap between what they feel and what the context requires them to display (Hochschild, 1983; Iseppato and Lupi, 2022). That burden is documented at every organizational level, and it diminishes Wellbeing (Vial and Cowgill, 2022). The three Triad capacities map onto this demand in ways that suggest a protective mechanism. Resilience supports adaptive emotion regulation strategies that may reduce the depleting cost of emotional labor, specifically by enabling emotional processing that does not create dissonance between felt and displayed emotion (Grandey, 2000; Mikolajczak et al., 2007). Proactivity allows women to act on emotional contexts rather than passively endure them, consistent with the agentic pathway described in the proactive motivation literature (Parker et al., 2010). Commitment sustains engagement with demanding work by making the effort feel self-determined rather than externally imposed (Deci and Ryan, 2000). When emotional labor is intrinsically motivated, it is less depleting than when it is externally demanded (Vial and Cowgill, 2022). Emotional labor does not disappear when the Triad is present. What changes is its cost.

For research and practice, the Triad offers a focused target. Rather than developing all 18 Brain Talents or even the 5 of the complete Resource Caravan, organizations can concentrate on 3 key emotional intelligence capacities that predict women's Wellbeing across job roles. The cumulative pattern suggests that even partial Triad development is linked to meaningful gains, while the full combination is associated with the strongest effect. The Resource Triad identifies which capacities warrant organizational investment, and evidence that EQ capacities can be enhanced through training interventions (Powell et al., 2024) suggests that organizational programs targeting Commitment, Resilience, and Proactivity may support women's Wellbeing at every career level.

### 4.4 The convergence effect: when the career-level gap narrows

The Resource Triad's relationship with Wellbeing holds across all four job roles, but its effect is not uniform. When Triad scores are low, career level shapes Wellbeing in expected ways. When Triad scores are high, those differences substantially narrow.

At zero of three Triad talents in the top tercile, the job role produces a clear stratification in Wellbeing outcomes. Only 17.4% of Students fall in the High Wellbeing tercile, compared to 23.4% of Management women and 24.6% of Senior Executives, a pattern consistent with the gain spiral dynamics described in Section 4.1 (Hobfoll, 1989; Hobfoll et al., 2018). When all three Triad talents are in the top tercile, the rank order shifts: Students and Senior Executives show the highest High Wellbeing rates (63.9% and 60.5%), with Employees and Managers slightly below (56.9% and 56.4%). The monotonic staircase is no longer present, and the Student-vs.-Senior-Executive gap that was highly significant at zero Triad talents (*z* = −5.61, *p* < 0.001) is no longer significant at three (*z* = 1.24, *p* = 0.21; Table 7). A 3.4 percentage-point gap remains (63.9% vs. 60.5%), but this is a fraction of the 7.2-point gap at zero Triad talents (17.4% vs. 24.6%). The influence of career level on Wellbeing substantially narrows rather than disappearing entirely.

The graded pattern reinforces the finding. Each additional Triad talent in the top tercile increases the likelihood of High Wellbeing (see Figure 2), and this gradient holds at every career level across the full 7-year study window. What changes is not the direction of the effect but its endpoint. As women accumulate Triad strengths, their Wellbeing outcomes converge regardless of where they sit in the organizational hierarchy.

COR's gain paradox principle offers an explanation. Hobfoll et al. (2018) observed that resource gain increases in salience in the context of resource loss. Women at earlier career stages face greater structural resource deficits: fewer mentors, less autonomy, and less organizational visibility. Yet those who have developed Commitment, Resilience, and Proactivity show Wellbeing outcomes comparable to women in senior positions operating in substantially richer condition-resource environments. This pattern is consistent with the gain paradox: the less the organization provides, the more these personal resources matter. Once the personal resource base is established, it is associated with Wellbeing regardless of organizational position. The emotional labor framework (Hochschild, 1983; Vial and Cowgill, 2022) suggests one reason this convergence may occur. When Commitment, Resilience, and Proactivity are well-developed, the ongoing cost of emotional labor is reduced, and career level loses its grip on Wellbeing outcomes.

The Student finding makes the gain paradox especially vivid. Among the four job roles, Students show the strongest Resource Triad effect (OR = 13.46, 95% CI [10.21, 17.74]), substantially exceeding the effect among workforce participants (OR = 7.29–8.44). Students occupy the lowest condition-resource position in this study. They lack the organizational supports, network access, and positional authority that accumulate with career advancement. Yet Students who have developed Commitment, Resilience, and Proactivity reach 63.9% High Wellbeing—the highest rate of any career level, surpassing even Senior Executives (60.5%). The less the environment provides, the more the personal resource base appears to carry.

Emerging qualitative research on women's resilience across career levels supports this pattern. Förster et al. (2025), studying female leaders in upper, middle, and lower leadership positions, found that the relative salience of individual resilience factors shifts with organizational position, with women in lower positions relying more heavily on personal capacities when institutional supports were scarce. The present findings offer large-sample quantitative evidence for a similar dynamic. In our data, when condition resources are sparse, personal emotional resources carry more of the Wellbeing load. When condition resources are abundant, the Triad still matters, but its marginal contribution is smaller because the organizational environment is already doing some of the protective work.

Industry data reflect the same pattern. McKinsey Company LeanIn.Org (2025) found that when workplaces are fair and inclusive, women are at least twice as likely to feel motivated, comfortable taking risks, and able to speak up, and significantly less likely to consider leaving. Deloitte (2025) reported that women with access to flexible working arrangements score 12 percentage points higher on mental wellbeing and are substantially more likely to want to stay with their employer long-term. When structural conditions deplete personal resources, Wellbeing and ambition decline together. Women who regularly work beyond contracted hours are far less likely to describe their mental wellbeing as good (15% vs. 58%) and far less likely to aspire to senior leadership (38% vs. 86%; Deloitte, 2025). In each case, when organizational conditions provide resource passageways (Hobfoll et al., 2018), they enable gain spirals: outcomes converge, just as the convergence effect in our data shows Wellbeing converging when personal resources are high.

For organizations, the convergence finding reframes the development question. Rather than asking women to prepare for the demands of each successive career level, these data suggest that organizations investing in Resource Triad development early, before workplace stressors accumulate, and this may yield disproportionate Wellbeing returns for women. The Student finding illustrates this potential. Women at the earliest career stage who scored in the top tercile on all three Triad capacities were over 13 times more likely to report High Wellbeing than those who did not. No other job role showed an association of this magnitude, suggesting that the potential return on organizational investment in EQ development may be greatest precisely where such investment has traditionally been lowest.

### 4.5 The squeezed middle: management vulnerability

Management women are the career level where the Resource Triad has declined most and delivers the smallest Wellbeing benefit. Their odds ratio of 7.29 is roughly half that of Student effect (OR = 13.46) and lower than both Employee (OR = 8.41) and Senior Executive (OR = 8.44) levels (see Figure 3). The Triad still matters for Management women, but it matters less than it does for women at every other career level.

Management women showed the largest Wellbeing decline among workforce participants (Δ = −1.57), while Employee Wellbeing declined more modestly and Senior Executive Wellbeing remained essentially stable over the study period (see Figure 4). Management women also showed the most sustained Brain Talent erosion, never recovering to 2019 levels (see Figure 5). This is not a temporary disruption. It is a sustained downward drift in the personal resources the Triad requires.

Management women are squeezed from above as they face increased demands at their career level including expanded accountability, the emotional labor that accompanies every interaction (Hochschild, 1983; Vial and Cowgill, 2022), and the visibility that comes with leading others. While job role demands are sufficient to deplete personal resources, Management women may lack the organizational supports that Senior Executives can access to replenish them. Research on women leaders confirms that these supports are critical for women to translate individual resilience into sustained performance (Förster et al., 2025). When those supports are absent, the translation fails.

In COR terms, this is a blocked resource passageway (Hobfoll et al., 2018; Son Hing et al., 2023). Industry data confirm how Management women experience this constraint from above. SHRM (2022) found that as women move from individual contributor to manager roles, their perception of organizational support deteriorates rather than improves. They report less encouragement to grow their careers, less inclusion in key networks, less access to internal promotion, and a growing belief that they are given fewer opportunities for upward career growth. The first promotion to manager remains the most persistent bottleneck in women's careers, with 93 women promoted for every 100 men, a ratio that has held for 11 consecutive years (McKinsey Company LeanIn.Org, 2025).

The squeeze also operates downward. Managers carry responsibility for their reports' development and wellbeing, taking on their teams' stress and frustration as part of the role. Yet they spend just 7% of their working hours on career development for their teams, citing lack of time and resources (McKinsey Company LeanIn.Org, 2025). The role is structured to demand more than it provides. The broader middle management picture confirms the pattern: 86% of managers report experiencing burnout, the highest rate of any organizational level, and nearly half indicated they were likely to quit within the year due to work-related stress (UKG Workforce Institute, 2023).

These are the organizational conditions, from above and below, that block the resource passageway for Management women. Where open passageways enable gain spirals, blocked passageways produce the opposite: resources erode without replenishment, and loss spirals accelerate.

For Management women in the middle, the Gallup data show what this looks like. Women managers report the highest burnout rate at 35%, coupled with 35% engagement (Barry and James, 2026). They are simultaneously the most effortful and the most depleted group in the workforce. A woman manager in this position is working hard, staying engaged, showing up for her team, and running out of capacity to keep going. Burnout research describes why this combination cannot hold: emotional exhaustion deepens when the resources that buffer against it are themselves consumed (Maslach and Leiter, 2016; Salvagioni et al., 2017), and chronic depletion erodes the very capacities required to reverse it (Mikolajczak et al., 2007).

COR theory predicts what follows. A woman manager running on depleted Commitment and Proactivity does not seek the next stretch assignment. She does not advocate for herself in a promotion conversation. She conserves what she has left. COR theory describes this shift from gain-oriented investment to defensive resource protection as a fundamental response to prolonged depletion (Hobfoll, 1998; Halbesleben et al., 2014). The Deloitte (2025) data suggest the shift is already widespread: women who regularly overwork are dramatically less likely to aspire to senior roles, at 38% compared to 86% of those who do not. This is not a lack of ambition. It is a natural response to sustained resource erosion, and it is itself a loss spiral: the depletion of resources reduces the very behaviors that could rebuild them.

The Emotional Recession compounds the squeeze. Freedman et al. (2025) documented sustained declines in EQ capacities across the global workforce from 2019 to 2024, identifying EQ as both a personal capacity and a collective organizational asset. For Management women, already positionally squeezed, this broader erosion thins the workplace conditions that could support recovery at the same time their personal resource base is shrinking.

The squeezed middle finding reframes where organizational investment matters most. Management women are not under-resourced in personal EQ capacities. The staircase pattern (see Figure 2) shows that their Brain Talent scores are higher than those of Employee women and Students. They are under-resourced in structural support. The demands of the role expand, but the supports do not keep pace. The management transition may function as a resource passageway that organizations can open or block by design. When managers actively support women's career development, women are far more likely to feel comfortable speaking up and taking risks (McKinsey Company LeanIn.Org, 2025). Formal mentoring, reduced administrative burden, protected time for people management, and recognition systems that reward developmental investment are not perks. In COR terms, they are the condition resources that replenish the resource base on which Wellbeing depends.

### 4.6 Women's workplace wellbeing as a modifiable dynamic

The four findings in this study point to a single dynamic. Five EQ capacities travel together and increase with career level (the Resource Caravan). Three of them, Commitment, Resilience, and Proactivity, capture nearly all of the Wellbeing prediction (the Resource Triad). When those three capacities are strong, career level's influence on Wellbeing substantially narrows, and the career-level staircase no longer holds (the Convergence Effect). And the career stage where those capacities are most depleted is where Wellbeing is most at risk (the Squeezed Middle). Taken together, these findings suggest that women's Wellbeing at work depends less on organizational position than on whether workplaces provide the resource pathways in which EQ capacities develop.

Evidence that EQ capacities can be developed through targeted intervention (Mattingly and Kraiger, 2019; Powell et al., 2024), including in organizational settings (Iseppato et al., 2024), suggests that the Wellbeing patterns documented in this study may be modifiable. The convergence finding is the strongest evidence for this prospect: women who have developed the Resource Triad reach comparable Wellbeing outcomes regardless of career level. The cumulative pattern means that scoring high on even one or two of the three Triad capacities predicts meaningful benefit.

Without organizational investment in these EQ capacities, women across the workforce face sustained erosion of the resources most strongly associated with Wellbeing, concentrated where the demands are greatest. This downward pressure, compounded by a broader Emotional Recession (Freedman et al., 2025), further erodes the workplace conditions in which recovery could occur. But the data also show the alternative. When Resource Triad scores are high, career levels' influence on Wellbeing substantially narrows. EQ capacities are measurable, they are learnable, and for women at every career level, they are the strongest predictors of higher Wellbeing.

### 4.7 Limitations and future directions

This study examines which specific EQ capacities predict women's Wellbeing across job roles. The core findings draw on a 7-year international dataset focused on behavioral applications of EQ. However, several limitations should be acknowledged.

The repeated cross-sectional design precludes causal inference. The career-level differences in Brain Talent scores could reflect selection effects (Schneider, 1987), developmental effects (Roberts et al., 2006), or both, and the present design cannot distinguish between them. The within-generation analysis partially addresses the age–job role confound but not the selection-vs.-development question. Future longitudinal studies tracking women across career transitions, particularly through the management level where vulnerability is greatest, could disentangle these mechanisms.

The reverse direction is equally plausible: women experiencing high Wellbeing may find it easier to exercise Commitment, Resilience, and Proactivity. COR theory anticipates this bidirectionality through its gain spiral principle (Hobfoll et al., 2018), but the data cannot resolve it.

Both Brain Talents and Wellbeing are measured by the same self-report instrument, the SEI, introducing the possibility of common-method variance (Podsakoff et al., 2003). A Harman's single-factor test (19.86% of variance on the first factor, 12 factors with eigenvalues ≥ 1) fell well below the threshold, indicating a single common factor, though Harman's test is a necessary but not sufficient check (Podsakoff et al., 2012). Converging evidence—distinct item sets, moderate bivariate correlations (*r* = 0.306–0.345), and cross-regional replication—provides additional mitigation (see Section 3.2). The SEI captures self-perceived emotional capacities rather than ability or behavior (Bar-On, 2006), and self-perception data can be influenced by response style and situational factors (Bru-Luna et al., 2021; D'Amico and Geraci, 2023). Future research could triangulate using multi-rater tools, behavioral observation (Boyatzis, 2018), or organizational outcome data.

The five Brain Talent predictors are highly intercorrelated, with the Imagination–Risk Tolerance pair producing severe VIFs (169.18, 169.77) due to near-perfect correlation (*r* = 0.982). For the Resource Triad, all VIFs were below 5 (maximum 4.62; Hair et al., 2010), and supplementary models confirm that Imagination and Risk Tolerance are statistically interchangeable (Supplementary Table S5). Given the collinearity in the full model, the study relied on bivariate correlations and odds ratios rather than regression (Tabachnick and Fidell, 2019). Future analytic approaches that accommodate correlated predictors could provide a finer decomposition of individual talent contributions.

Several dimensions fall outside the present measurement scope. Job role is self-classified into four broad categories that encompass meaningfully different structural realities. The SEI does not measure organizational conditions such as mentoring access, autonomy, or psychological safety. This study therefore measures how personal capacities predict Wellbeing but not the organizational conditions that COR theory predicts enable those capacities; the Discussion triangulated with large-scale industry data to provide indirect support, but future work should measure both simultaneously. The dataset does not include race, ethnicity, socioeconomic class, caregiving status, disability status, or sexuality, and the analysis cannot examine patterns across intersectional subgroups (Stamarski and Son Hing, 2015). Independent criterion validation of the SEI Wellbeing subscale against established measures (e.g., WHO-5) would strengthen the interpretive basis. Future research addressing these gaps could test whether the Triad's association with Wellbeing holds across women's diverse structural positions and measurement approaches.

The SEI dataset comprises individuals assessed in applied practice settings. Many completed the SEI as part of organizational requirements rather than self-selecting for interest in EQ, but the sample context may influence mean scores relative to the broader workforce. The consistency of findings across 7 years, three regions, and a within-generation replication partially mitigates this concern. Formal measurement invariance testing across language groups would further strengthen the cross-regional findings. This study examined women exclusively, responding to the special issue focus; future studies could test whether the Resource Triad operates similarly across genders.

Beyond these directions, intervention studies could test whether targeted Triad development produces the Wellbeing gains that the observational data imply. Whether emotional labor mediates the Triad–Wellbeing relationship is a testable question. The convergence finding warrants replication with longitudinal data to determine whether it reflects a genuine pattern or a restriction-of-range artifact. Although the Resource Triad effect was stable across two non-overlapping time periods, annual odds ratio analyses could provide additional granularity on temporal trends.

This study identifies a small set of EQ capacities that predict women's Wellbeing with consistency across career levels, regions, and generations. Whether those capacities can be built through intervention, whether the organizational conditions that support them can be measured and changed, and whether the convergence effect holds when tested prospectively are the questions that will determine whether these findings translate from pattern to practice.

## Data Availability

The datasets presented in this article are not readily available because the data analyzed in this study is subject to the following licenses/restrictions. The dataset analyzed in this study is derived from the SEI emotional intelligence assessment, which is the proprietary instrument of Six Seconds. Because of privacy, licensing, and organizational confidentiality restrictions, the raw data cannot be shared publicly. Researchers interested in collaboration or secondary analyses may contact Six Seconds to discuss data access under appropriate agreements. Requests to access the datasets should be directed to daniel.choi@6seconds.org.

## Supplementary material

Supplementary Table S1 Definitions of the 18 SEI brain talents.

Supplementary Table S2 Intercorrelations among the five resource caravan brain talents and wellbeing.

Supplementary Table S3 Confidence interval values for all figure data points.

Supplementary Table S4 Odds ratios for all ten three-talent combinations from the five resource caravan brain talents.

Supplementary Table S5 Collinearity diagnostics: variance inflation factors, tolerance, and *R*^2^ for the full five-predictor, drop-one, and resource Triad models, with zero-order correlation matrix.

Supplementary Table F1 Generative AI usage documentation.
