# Supplementary material for: Into the headwinds: key emotional intelligence capacities that predict women's workplace wellbeing across job roles
Source: Front Psychol. 2026 May 29;17:1848879. doi: 10.3389/fpsyg.2026.1848879 (PMC13261904; doi:10.3389/fpsyg.2026.1848879)
Supplement: Supplementary Table F1 — Generative AI usage documentation. [file Supplementary_file_1.docx]

**Supplementary File: Generative AI Prompts and Outputs Used in Manuscript Preparation**

**Purpose of this Supplementary File**

This file documents the use of generative artificial intelligence (Claude Opus 4, Anthropic, 2026) in the preparation of this manuscript, in compliance with *Frontiers* editorial policy. It includes representative prompts submitted to the AI tool and the corresponding outputs that informed manuscript editing and formatting. Additional exchanges followed the same procedures and were reviewed, edited, and verified by the authors.

All AI-generated material was reviewed, edited, and verified by the authors for accuracy, originality, and relevance. Generative AI was not used for data collection, statistical analysis, or interpretation of results. No generative AI tool is listed as an author, and the authors accept full responsibility for the scientific and editorial content of the manuscript.

**Section 1: Editorial Revision and Repetition Reduction**

**Task:** Identifying and reducing proximity repetition across the manuscript.

**Prompt:** “Please identify instances where the phrase ‘suggests that’ appears within a few lines of each other in the Discussion section, and propose varied alternatives that maintain the same meaning.”

**Output:** Returned a list of instances with proposed replacements (e.g., “indicates,” “points to,” “signals”).

**Usage:** Authors reviewed each proposed replacement individually, accepted or rejected alternatives based on meaning and academic tone, and verified that no new proximity repetitions were introduced. AI identified patterns; all editorial decisions were made by the authors.

**Section 2: Formatting and Style Compliance**

**Task:** Standardizing reference formatting and section numbering to Frontiers style.

**Prompt:** “Please standardize the section numbering in the Discussion to match the format used in the Methods and Results sections.”

**Output:** Identified inconsistencies (e.g., colons in some subsection headings, period in main heading) and proposed corrections.

**Usage:** Authors verified corrections and applied them. AI support was limited to identifying formatting inconsistencies; all substantive decisions were made by the authors.

**Section 3: Abstract Word Count Reduction**

**Task:** Condensing the abstract to meet Frontiers word limits.

**Prompt:** “Please identify redundant phrases and suggest shorter alternatives to reduce the word count of this abstract while preserving the key statistics.”

**Output:** Returned a list of phrases with shorter alternatives.

**Usage:** Authors selected which phrases to shorten and which to retain, verified that all key data points remained, and wrote the final version. AI identified candidates for compression; all decisions about what to keep or cut were made by the authors.

**Section 4: Keyword Optimization for Discoverability**

**Task:** Evaluating keyword choices for academic search engine optimization.

**Prompt:** “Evaluate our current keywords against the special edition call for papers and suggest alternatives with higher search volume.”

**Output:** Generated candidate keywords and suggested reordering by search volume.

**Usage:** Authors conducted independent search volume verification using Google Scholar, selected final keywords based on combined relevance and discoverability, and determined the final ordering. AI generated initial suggestions; all final keyword selections were made by the authors.

**Section 5: Causal Language Audit**

**Task:** Reviewing manuscript for language that implies causation beyond what the cross-sectional design supports.

**Prompt:** “Audit the Discussion section for causal language that implies directionality. Flag terms like ‘protect,’ ‘buffer,’ ‘drive,’ ‘produce,’ and ‘eliminate’ and assess whether each use is appropriate for a cross-sectional design.”

**Output:** Returned a categorized list of instances with assessments of whether each was problematic, acceptable (e.g., within COR theory framing), or borderline.

**Usage:** Authors reviewed all flagged instances, accepted or rejected each proposed change, and determined which theoretical uses of causal language were appropriate within the COR framework. AI identified patterns; all interpretive and editorial decisions were made by the authors.

**Author Verification**

All outputs were reviewed, edited, and verified by the human authors to ensure factual accuracy, relevance, and alignment with the study’s intent. No AI tools were used for data analysis, interpretation of results, or generation of theoretical arguments. Claude Opus 4.6 is not listed as an author.

**Model Information**

- **Name:** Claude
- **Model:** Opus 4 (Anthropic)
- **Access period:** January–April 2026
- **Source:** https://claude.ai
